# Supporting people with dementia and their carers in rural Kenya. Emerging evidence from the ENGAGE project

**DOI:** 10.1002/alz70860_098188

**Published:** 2025-12-23

**Authors:** Purity Mwendwa, Andrew Clark, Iracema Leroi

**Affiliations:** ^1^ Trinity College Institute of Neuroscience, Trinity College Dublin, Dublin, Ireland; ^2^ Institute for Lifecourse Development, University of Greenwich, London, United Kingdom

## Abstract

**Background:**

In African countries, most people with dementia are cared for at home, but limited knowledge about dementia, lack of diagnosis and misdiagnosis and no formal supports leaves families with sole caring responsibilities. Limited knowledge leads to stigma and potential marginalization of those affected. This qualitative study examines the support needs of people with dementia and those who care for them at home in rural Kenya.

**Methods:**

The study was part of a wider study, ENGAGE, and used semi‐structured interviews to gather data. We interviewed 17 people including people living with dementia, carers, health care workers and community members in Meru County. Participants were aged between 42 and 88 years and included 12 females and 5 males. Data were analysed using thematic analysis and findings were structured based on 4 of the Alzheimer Scotland 8 Pillar Model of Community Support namely; personalised support, support for carers, general healthcare and treatment and community connections.

**Results:**

There is a huge knowledge gap and burden associated with caring for people living with dementia in rural Kenya. The lack/limited knowledge about dementia among family members, carers and health care workers was highlighted. Educating these individuals about dementia and equipping them with caregiver skills was considered vital to ease burden and address misconceptions about dementia. The role of the national and county government in developing training for carers and health workers was emphasised and so was the need for governments to build care homes for those at advanced stages of dementia and those with no family support. Participants called on the government to provide personalised support (finances and health insurance cover) to families affected. Religious institutions, such as the church were seen as having a moral responsibility to ensure that people with dementia and their families are not excluded from the community.

**Conclusion:**

With the number of people living with dementia projected to increase in Kenya, addressing the support needs of those affected is urgent. This study comes at an opportune time when the Ministry of Health in Kenya is developing a dementia policy, and findings from this study can inform the development of this policy.

